# Public perceptions of international genetic information sharing for biomedical research in China: a case study of the social media debate on the article “A Pangenome Reference of 36 Chinese Populations” published in *Nature*

**DOI:** 10.1186/s40246-024-00650-4

**Published:** 2024-08-07

**Authors:** Zhangyu Wang, Meng Wang, Li Du

**Affiliations:** Faculty of Law, University of Macau, E32, Avenida da Universidade, Taipa, Macau China

**Keywords:** Biosecurity, Chinese gene-sequencing data, International genetic data sharing, Biomedical research, Cross-border genomics research collaboration, Public attitudes, Public education, Law knowledge, Legal regulation, Collective culture

## Abstract

**Background:**

The international disclosure of Chinese human genetic data continues to be a contentious issue in China, generating public debates in both traditional and social media channels. Concerns have intensified after Chinese scientists’ research on pangenome data was published in the prestigious journal Nature.

**Methods:**

This study scrutinized microblogs posted on Weibo, a popular Chinese social media site, in the two months immediately following the publication (June 14, 2023–August 21, 2023). Content analysis was conducted to assess the nature of public responses, justifications for positive or negative attitudes, and the users’ overall knowledge of how Chinese human genetic information is regulated and managed in China.

**Results:**

Weibo users displayed contrasting attitudes towards the article’s public disclose of pangenome research data, with 18% positive, 64% negative, and 18% neutral. Positive attitudes came primarily from verified government and media accounts, which praised the publication. In contrast, negative attitudes originated from individual users who were concerned about national security and health risks and often believed that the researchers have betrayed China. The benefits of data sharing highlighted in the commentaries included advancements in disease research and scientific progress. Approximately 16% of the microblogs indicated that Weibo users had misunderstood existing regulations and laws governing data sharing and stewardship.

**Conclusions:**

Based on the predominantly negative public attitudes toward scientific data sharing established by our study, we recommend enhanced outreach by scientists and scientific institutions to increase the public understanding of developments in genetic research, international data sharing, and associated regulations. Additionally, governmental agencies can alleviate public fears and concerns by being more transparent about their security reviews of international collaborative research involving Chinese human genetic data and its cross-border transfer.

## Background

In mid-June 2023, a group of Chinese genomics scholars announced a landmark achievement—the publication of the first pangenome reference in the prestigious scientific journal *Nature* (hereinafter referred to as “the Publication Activity”). The research involved a relatively wide sampling, incorporating “116 high-quality de novo assemblies from 58 core samples representing 36 Chinese ethnic groups and 6 assemblies of the Han Chinese majority [1].” Its impact was substantial in improving the less represented genomic information of populations of Asian ancestry, for its identification of “15.9 million small variants and 78,072 structural variants, of which 5.9 million small variants and 34,223 structural variants were not reported in a recently released pangenome reference [1].” This accomplishment symbolizes the first research milestone in the genomics field, independently completed by Chinese scientists [1]. Numerous news reports have lauded this research as “a historic leap in China’s genomics over the past 40 years,” due to its substantial representation of the vast genomic profiles of Chinese population [2]. This achievement is believed to have great potential for efforts to improve the prevention and treatment of genetic diseases among Chinese people, support the implementation of China’s health policies, and advance knowledge of genomics in medicine [2, 3].

However, some dissenting opinions have highlighted potential risks associated with the international publication of this research. Critics have argued that the pangenome reference could contain all potential variant sequences of Chinese population [4]. As such, they believe the public dissemination of Chinese genomic sequences could pose substantial national biosecurity risks, thereby warranting the intervention of the Chinese national security authority for further investigation and management [5, 6]. Specifically, opponents of the Publication Activity highlights the possibility of aiding foreign adversaries in developing biological and genetic weapons targeted at the Chinese population. This could make Chinese people more susceptible to certain bacteria and viruses, specifically cultivated or engineered using the disclosed genetic information [7]. Moreover, the *Implementing Rules for the Administrative Regulations on Human Genetic Resources* (hereinafter referred to as “the Implementing Rules”) that came into effect shortly after the Publication Activity may have fueled suspicions and concerns about the real purpose of the publication, as many commentators speculate that the timing of the Publication Activity was not coincidental, but rather a deliberate attempt at circumventing the *Implementing Rules* [7].

Previous research has demonstrated that diminished public trust could detrimentally impact the acquisition and sharing of genetic information, as well as biomedical research endeavors in principle [8, 9]. Moreover, negative public sentiments could also adversely affect governmental support for certain research initiatives or industries, especially the prioritization of research funding allocation and collaborations between industry and policymakers [10, 11]. Notably, escalating public distrust and resistance could potentially lead to more restrictive legislation that hampers the cross-border genetic data transfer, subsequently reducing scientists’ involvement in global research collaborations [12]. Instances of such legislative interventions are not uncommon in China. In the 1997 Anhui incident, for example, two epidemiologists from Harvard University took blood samples from over 16,000 farmers in Anhui Province, China, without appropriately obtaining their consents. The incident sparked public concerns about the malicious use of genetic resources, and the ensuing public backlash hastened the promulgation of the *1998 Interim Measures for the Administration of Human Genetic Resources* one year later [13, 14]. The ethical controversy over He Jiankui’s work on the first gene-edited babies in 2018 is another example of how widespread public concern and criticism led to the criminalization of illegally implanting gene-edited and cloned embryos in the 2020 amendment to China’s *Criminal Law* [15].

Recent studies have examined different aspects of the Chinese regulatory framework for managing human genomic information, including the collection, use, and cross-border transfer of genetic data. For instance, there are concerns that the existing legal requirements in China for regulating cross-border genetic data transfer and managing human genetic resources are considerably strict [16]. The current legal context in China is in stark contrast with international regulatory developments that emphasize open genetic data sharing, such as the United Kingdom’s receptiveness to global scientists using its domestic public biobanks and consistent improvements in health data governance [17]. There is still a scarcity of research on the public perceptions of international genetic data sharing for research purposes in China. In 2020, the Global Alliance for Genomics and Health conducted a global survey involving 3,008 participants from China, posing 29 questions regarding their willingness to donate genomic data, familiarity with genomics, trust in data users, and related issues. The survey revealed a strikingly low public acceptance of donating genomic data for research use [18]. However, this study did not explore the reasons behind the generally negative public attitudes, nor did it examine the public understandings of regulations related to Chinese human genetic data sharing.

The public debate stirred by the Publication Activity in *Nature* presented a good case study to examine public perceptions on genetic data sharing for biomedical research purposes. We used Sina Weibo, a popular social media platform in China, to observe public comments and discussions about the publication of Chinese human gene-sequencing data. Given Weibo’s expansive user base, including 511 million active users according to statistical data as of September 2020, diverse age groups are engaged in daily discussions on social hotspots in China [19]. Discussions on this microblogging platform can provide insight into the public perceptions of human genetic data sharing. In this study, we investigate how members of the public discussed the Publication Activity in *Nature* and identify the reasons behind their attitudes. In addition, we highlight the importance of increasing the public understanding of genomic-based biomedical research, and especially issues concerning biosecurity and the regulatory regime for Chinese human genetic information.

## Methods

We initially used the Weibo search box to conduct an exploratory search for identifying suitable keywords for our formal data search and collection. Several categories were then determined, including the official Chinese translation of the research’s title (A Pangenome Reference of 36 Chinese Populations (基于36个族群的中国人泛基因组参考图谱), and the names of corresponding authors and their academic affiliations—(Shuhua Xu (徐书华), Kai Ye (叶凯), Jiayou Chu (褚嘉祐), Yan Lu (陆艳), Fudan University (复旦大学), Xi’an Jiaotong University (西安交通大学), and Chinese Academy of Medical Sciences (中国医学科学院)). For more search accuracy, we used the advanced settings of the search box to retrieve each author and affiliation with the qualifier of “gene” (in Chinese: “基因”) and “genetic information” (in Chinese: “遗传信息”), respectively. We limited the search period from June 14, 2023, when the research paper was published online, to August 21, 2023, which was the cut-off date for data collection. The retrieval originally generated 968 microblogs in Chinese. Subsequently, preliminary analysis was conducted to exclude microblogs that were reduplicative and irrelevant to our topic. The final dataset for our study contained 306 microblogs.

The content analysis we conducted comprised two phases. Initially, applying methods previously developed by our research team [20], we first selected 10% of dataset, and completed an exploratory thematic analysis to develop a coding framework [12, 21]. Subsequently, one of the authors of the study independently coded the dataset based on the following coding questions: (1) What is the identity of the Weibo user? (This identity should be certified by Weibo); (2) How popular is the microblog?; (3) Does the poster share relevant scientific knowledge of genomic-based research?; (4) What is the attitude shown in the microblog towards the Publication Activity?; (5) If the attitude is positive, what reasons are mentioned by the microblog?; (6) If the attitude is negative, what reasons are mentioned by the microblog?; (7) What risks associated with public disclosure of genetic sequence are mentioned in the microblog?; (8) What benefits associated with public disclosure of genetic sequence are mentioned in the microblog?; (9) Does the microblog mention regulations on genetic data sharing?; and (10) Does the microblog mention that the researchers (the authors and/or universities) should be held liable?

In assessing the attitudes conveyed in microblogs, the coder identified microblogs that merely reposted other people’s microblogs without adding any their own original comments as having unclear attitude. As such, the coder categorized these microblogs as “showing no clear attitude.” The rationale for including this kind of microblogs into the dataset and making such categorization were that the reposting activities signified Weibo users were concerned about the event, while it was uncertain whether their attitudes were in line with the content they reposted, given that they themselves did not provide any textual commentary on the Publication Activity to enable a more precise analysis. To determine the popularity of microblogs, we developed criteria based on an initial exploratory search. This was accomplished by the number of “Likes,” “Comments,” and “Reposts” each post garnered. Specifically, microblogs with over 500 likes were classified as “Highly Liked,” those receiving more than 500 comments were designated as “Heated Comment,” and microblogs with over 500 reposts were categorized as “Highly Reposted.”

To minimize potential interpretive biases and assess the reliability of the coding, an independent coder randomly selected and analyzed 30% of the dataset using the same analytical scheme. During this process, the main disagreement between two coders was on the question “What is the reason(s) for negatively viewing the Publication Activity mentioned in the post?.” After discussion, two coders unanimously agreed that only explicit statements in the posts, such as “this Publication Activity leaking genetic information is betrayal, selling out the country,” “this is collusion with foreign forces,” or “the researchers are spies from other countries,” signified the posters’ beliefs about the Publication Activity as involving “betrayal to China.” Thus, a mere critique of the research being published in foreign journal or researchers being xenocentric did not equate to claims about “betrayal to China.” We next conducted an interrater reliability assessment using Cohen’s Kappa (K) to establish the rate of agreement between the two coders. Based on Landis and Koch’s benchmark for interpreting kappa, our coding results demonstrated almost perfect agreement scores (K = 0.855–1.000) on all coding categories [22].

## Results

**Fig. 1:**
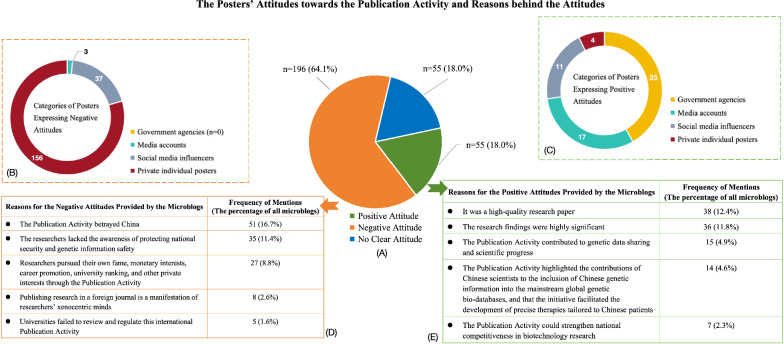
The Posters’ Attitudes towards the Publication Activity and Reasons behind the Attitudes. **A** Weibo posters’ different attitudes towards the Publication Activity involving genetic information disclosure. **B** Categories and corresponding numbers of posters expressing negative attitudes. **C** Categories and corresponding numbers of posters expressing positive attitudes. **D** Reasons for negative attitudes and corresponding frequency of mentions. **E** Reasons for positive attitudes and corresponding frequency of mentions

Our analysis indicated that 18.0% of microblogs (n = 55) viewed the Publication Activity positively (See Fig. 1). These microblogs typically praised the Publication Activity as a research breakthrough or considered it beneficial for biomedical research and innovation without any criticism. By contrast, 64.1% of the social media posts (n = 196) held negative attitudes. These microblogs typically criticized the Publication Activity or questioned the motives behind the release of genomic sequencing data (See Fig. 1). The remaining microblogs (n = 55, 18.0%) merely reported the event and did not show any attitudes (See Fig. 1).

In terms of the background of users, 24 microblogs were posted by government agencies (7.8%), 20 were from media sources (6.5%), and 52 were written by social media influencers (SMI) (17.0%) that had been authenticated by Weibo. The rest of the microblogs came from private individual accounts (n = 210, 68.6%). Among microblogs with positive attitudes, 92.7% (n = 51) came from verified government, media, and SMI accounts, while 7.3% (n = 4) were from non-authenticated private individuals (Fig. 1). There were no microblogs with negative attitudes from government accounts. Conversely, 79.6% of microblogs with negative attitudes (n = 156) were posted by private individual posters (Fig. 1).

Six microblogs (2%) provided scientific explanations or explained scientific notions related to genomic-based research and innovation, and none of them was posted by users with medical or scientific and technological background. In terms of the popularity of the post, only two microblogs with positive attitudes matched the popularity criterion with being defined as “Highly Liked.” One of them came from the authenticated media account of the Technology Channel of China Central Television, and the other one from an SMI account. Thirteen microblogs (4.2%) expressing negative attitudes satisfied the criterion of popularity, with all of them being recognized as “Highly Liked,” three of them as “Highly Reposted,” and three of them as “Heated Comment.” Among these 13 microblogs, two microblogs—one from an authenticated media account (personal media) and the other one from an SMI (an online writer)—concurrently met the three criteria of popularity. Both received over 10,000 Likes.

We established that 53 microblogs indicated specific reasons for their positive attitudes (17.3%) (Fig. 1). The high quality of the research recognized by *Nature* was mentioned in 38 microblogs, and 36 microblogs generally acknowledged the significance of the research results. Fifteen microblogs (4.9%) held that the Publication Activity contributed to genetic data sharing and scientific progress in the field. Fourteen microblogs (4.6%) asserted that the Publication Activity highlighted the contributions of Chinese scientists to the inclusion of Chinese genetic information into the mainstream global genetic bio-databases and that the initiative would facilitate the development of precise therapies tailored to Chinese patients. Seven posters (2.3%) claimed that the Publication Activity could strengthen national competitiveness in biotechnology research.

Furthermore, 77 microblogs with negative attitudes (25.2%) provided reasons against the Publication Activity (Fig. 1). The top three reasons were: (1) that “such Publication Activity betrayed China” (n = 51, 16.7%); (2) that “the researchers lacked the awareness of protecting national security and genetic information safety” (n = 35, 11.4%); and (3) that “researchers pursued their own fame, monetary interests, career promotion, university ranking, and other private interests through the Publication Activity” (n = 27, 8.8%). Additionally, eight microblogs (2.6%) viewed publishing research in a foreign journal as a manifestation of researchers’ xenocentric minds, while another five microblogs (1.6%) condemned the universities’ failure in reviewing and regulating this international Publication Activity.

Benefits from publishing Chinese human genetic information were only mentioned in 4.9% of the microblogs (n = 15) (Table 1).

**Table 1:** Benefits and risks of publishing Chinese human genetic sequencing data portrayed in microblogs

Benefits	Frequency of mentions (the percentage of all microblogs mentioning benefits)
• Contributing to biomedical research and the development of disease research and treatments customized for Chinese patients	14 (93.3%)
• Facilitating scientific progress in genomic research	12 (80.0%)
• Promoting the development of China’s healthcare industry and policy implementation	8 (53.3%)

Risks	Frequency of mentions (the percentage of all microblogs mentioning risks)
• Hostile countries’ biological or genetic weapons development against Chinese people	77 (79.4%)
• Foreign countries’ manipulation and abuse of Chinese genetic information, consequently introducing threats to health and life of Chinese people	43 (44.3%)
• Threats to China’s national (bio)security	31 (32.0%)
• Threats to the safety of foreign civilians of Chinese ancestry or the Chinese diaspora living outside China in the event of a genetic information leak	10 (10.3%)
• Privacy breaches due to genetic information disclosure	6 (6.2%)

Among these, 14 microblogs (93.3%) praised its positive impact on biomedical research and the development of disease research and treatments customized for Chinese patients. Furthermore, 12 microblogs (80.0%) emphasized that the publication of Chinese genetic data indicated China’s scientific progress in genomic research and another eight (53.3%) highlighted its impact on the development of China’s healthcare industry and policy implementation. By contrast, 31.7% (n = 97) expressed concerns about the risks of publishing Chinese human genetic information (See Table 1). Among all concerns, the three main ones included: (1) that such information disclosure may result in “hostile countries’ biological or genetic weapons development against Chinese people” (n = 77, 79.4%); (2) fears about “foreign countries’ manipulation and abuse of Chinese genetic information, consequently introducing threats to health and life of Chinese people” (n = 43, 44.3%); and (3) “threats to China’s national (bio)security” (n = 31, 32.0%). Furthermore, 10 microblogs (10.3%) expressed worries about the safety of foreign civilians of Chinese ancestry or the Chinese diaspora living outside China in the event of a genetic information leak. Additionally, concerns about privacy breaches due to genetic information disclosure were raised in six microblogs (6.2%).

Our analysis showed that 50 microblogs (16.3%) mentioned regulatory approaches to human genetic resources. For instance, 34 Weibo users argued that the timing of the Publication Activity, which was released just before the introduction of the new *Implementing Rules*, was a deliberate attempt to circumvent the law. There were 13 microblogs (4.2%) which questioned whether the Publication Activity had been reviewed and approved by the appropriate authorities. However, one microblog asserted that the Publication Activity and associated genetic data sharing activities had been approved by China’s Ministry of Science and Technology (hereinafter referred to as “MOST”). Nonetheless, two microblogs alleged that the Publication Activity had violated the China’s *Biosecurity Law*, and 32 Weibo users (10.5%) called for an investigation into and punishment for the Publication Activity. Seven microblogs (2.3%) held the universities of the researchers liable for the Publication Activity, six of which explicitly urged the universities to provide clarification or/and commitments that the Publication Activity would not pose risks to national security or human health. One post argued that all Chinese people were entitled to sue the respective universities and the researchers involved for compensation.

## Discussion

In this study, we provide a close examination of Weibo users’ comments on the publication of Chinese gene-sequencing data in a prestigious international scientific journal, including their attitudes towards this Publication Activity, reasons behind their opinions, and laypersons’ perceptions regarding the protection of human genetic resources. We have identified several major considerations emerging from our findings that are relevant to and could potentially impact the reform of Chinese human genetic information management in China.

### Divergent perspectives between government and individual users

An important finding is that authenticated government accounts did not express any negative attitudes toward the Publication Activity. The positive perspectives signify the Chinese government’s willingness to share genetic information and their endorsement of the principle of open science. Conversely, microblogs from individual Weibo users typically exhibited more negative attitudes towards the Publication Activity, primarily motivated by concerns that the disclosure of genetic information could potentially jeopardize Chinese national security. This discrepancy suggests that the public may have misconceptions about the sharing of human genetic data. There are two major reasons that may account for these negative public attitudes. First, the distinct value of genetic information has been defined by the established laws. For instance, the 2021 *Personal Information Protection Law* (hereinafter referred to as “the PIPL”) categorized genetic information as sensitive data, and the 2021 *Biosecurity Law* regulated it as an essential element in preserving national security. Secondly, amidst the COVID-19 pandemic, there were popular beliefs that SARS-CoV-2 was purposely developed by the West to target the genetics of the Chinese population [23, 24]. This may have amplified public distrust and dissent on issues concerning the international publication of Chinese human genetic information. Consequently, our study emphasizes the harmful influence of misinformation on the public’s trust in scientists.

### Impact of a culture emphasizing collective interests

In our analysis, Weibo users with negative attitudes preferred to identify themselves as parts of the Chinese population, rather than as individuals potentially affected by the data disclosure in the Publication Activity. As demonstrated by microblogs, less than 2% of microblogs (n = 6) discussed the issue of personal data protection and privacy violations that the Publication Activity may bring. Previous analyses of Chinese legal culture have indicated that the preservation of individual privacy in China should consider the “collective interests of the nation and society, equitable distribution of benefits from data flow among social actors, and special protection for vulnerable groups, to maintain legal and moral integrity [25, 26].” Therefore, Weibo users’ tendency to downplay individual privacy and personal information safety while prioritizing national security, is consistent with traditional Chinese values and approaches shaped by a collectivist culture [27–29]. More specifically, the prevailing collectivist mindset typically heightens Weibo users’ sense of solidarity and their concerns about national security risks to the entire Chinese population, often prioritizing such considerations over individual privacy and information sharing.

### Knowledge gaps regarding the regulation of genetic information

Our research indicates that Weibo users may have limited knowledge of how genetic information is regulated in China. For instance, cross-border genetic data transfer is primarily governed by both the PIPL and *the Administrative Regulations on Human Genetic Resources* (hereinafter referred to as “HGR Regulations”), but no microblogs mentioned these laws when discussed legal issues related to the Publication Activity. Moreover, laypersons’ inaccurate perceptions of the regulatory context could be the driver of their negative attitudes. For example, 68% of all microblogs containing legal appraisals suggested that the Publication Activity intended to evade the newly introduced *Implementing Rules*. However, contrary to public perceptions, the *Implementing Rules* did not establish new conditions for international collaborations using Chinese human genetic sources; rather, it merely explained situations that could trigger security review by the MOST, a legal regime that had already been outlined in the *Biosecurity Law* and HGR Regulations. Besides, institutions associated with the Publication Activity, such as Fudan University, clearly stated on their official websites that the research and associated genetic data sharing had been approved by the MOST. However, the approval from the MOST was rarely mentioned by Weibo users, with merely one microblog stating this procedure.

Additionally, only one Weibo user from the legal sector, authenticated by Weibo as an SMI (a deputy director at a Judges’ Training Institute), participated in the discussion. Nevertheless, the microblog did not present legal arguments, but rather speculated on the connection between genetic information disclosure and bioweapons and biowarfare. The limited participation of legal professionals in social media discussions on such issues is not helpful for correcting widespread misconceptions about regulations on human genetic resources. Without a timely response from legal experts, misconceptions about the disclosure of human genetic information are likely to shape the public debate on Weibo.

The *Biosecurity Law* mandates government agencies, universities, and news media to publicize and popularize biosecurity laws, regulations, and general knowledge of biosecurity [30]. However, gaps in laypersons’ knowledge concerning the regulatory environment for genetic information, established by our study, suggest that governmental agencies, official media, and research institutions have not efficiently communicated accurate biosecurity and legal knowledge to the public.

### Advocating for a better public education on biosecurity and related legal issues

Strong negative public attitudes towards genetic data sharing could exert pressure on scientists in the process of their research work and the development of research outputs. Public opposition could also impact the enforcement of laws and policies related to genetic data sharing and biotechnology research, thereby impeding international research collaborations that legitimately utilize China’s genetic data resources. In the long run, such biased public attitudes can have lasting influence on framing new legal regulations and policies adaptive to societal needs and development, since the power of public sentiments, as we mentioned earlier in the Anhui incident and He Jiankui gene-editing case, fairly amounts to be a driving force [31].

Our analysis highlights the need for future efforts by researchers, media, and governments to increase scientific literacy and better educate the public about benefits and risks associated with biomedical research utilizing human genetic information. Previous studies have demonstrated that after research participants learned more about biomedical research, their trust in biobanks and willingness to donate individual biospecimens increased significantly [32]. Sound public education would enable the public to accurately understand legitimate research activities while remaining alert to unauthorized ones [32–34]. It would also encourage their engagement in more research initiatives, fostering a favourable research environment. Educational outreach would prepare the public to provide insightful comments and recommendations on future legislations and policies regarding genetic data sharing. This would prevent misunderstandings and calm public fears which have the potential to further exacerbate China’s already stringent legal regimes. Ultimately, negative public attitudes could impede the development of a more permissive and more responsible legal framework for international sharing of genetic data for research purposes.

In the present case, the announcement concerning the research authorization and legal compliance for publishing human gene-sequencing data could have been clearly articulated on the official websites of Fudan University and Xi’an Jiaotong University [35]. It would also have been helpful if official statements from governmental agencies or research institutions were promptly issued to dispel widespread public scepticism and concerns. As a further step, in order to encourage positive and supportive public attitudes towards genetic data sharing and biomedical research, it would be practical to establish an official platform to publicize information about authorized research projects. This could include best practices in genetic data sharing that comply with the regulatory requirements. Detailed guidelines for public communication could enhance the performance of this official platform.

## Conclusion

Our study revealed a clear discrepancy in attitudes towards the Publication Activity between governmental actors and individual users on Weibo. All microblogs posted by official governmental accounts displayed positivity and support for the publication of Chinese human gene-sequencing data, while individual users generally held negative perceptions about the Publication Activity. Such negative public attitudes primarily originate from perceptions about risks to national security and public health that may result from the international exposure of Chinese genetic information to potential hostile entities. China’s collectivistic culture reinforces these worries about national security and public health. These factors have likely fuelled the public’s increasingly negative attitudes towards genetic data sharing in international collaborative biomedical research.

Gaps in public knowledge and misperceptions about laws relating to genetic resource management suggest that enhanced educational outreach and programs are needed to improve the public understanding of genetic research and international genetic data sharing. It would be beneficial if the MOST adopts a more transparent regime for security review concerning the use of Chinese human genetic resources in international collaborative research and the provision of such resources to foreign entities. Furthermore, scientists and legal scholars could more actively engage in Weibo debates. Their input is crucial for addressing misinformation in a timely manner and for increasing the public understanding of scientific and legal issues associated with the disclosure of human genetic information.

## Limitations of the study

This study has two major limitations. First, it is primarily focused on the public’s initial reactions following the publication of the research, rather than on the potential long-term impact of public attitudes on policymaking, scientific collaboration, or public health initiatives. Future retrospective research could contribute to a more comprehensive understanding of the long-term impact. Second, while we observed a significant portion of microblogs holding misconceptions towards China’s legal regulation on genetic data management, we did not include an in-depth investigation into whether these misconceptions could be effectively addressed through the proposed solutions. Further research is needed to explore the effectiveness of the solutions we recommended, such as educational strategies and measures to increase governmental transparency. Despite these limitations, our study offers valuable insights into the Chinese public perceptions of international genetic information sharing for biomedical research, and would timely inform government agencies, both Chinese and international scientific communities, and other stakeholders about potential public concerns surrounding future biomedical research utilizing genetic data originated from China.

## Data Availability

In compliance with the Personal Information Protection Law of the People’s Republic of China, the dataset used and analyzed in the study is protected and not openly available. However, it can be made accessible upon a reasonable request to the corresponding author.

## Abbreviations

MOST: The Ministry of Science and Technology of the People’s Republic of China
PIPL: Personal Information Protection Law of the People’s Republic of China
SMI: Social media influencers
